# Interactive Effects of Large- and Small-Scale Sources of Feral Honey-Bees for Sunflower in the Argentine Pampas

**DOI:** 10.1371/journal.pone.0030968

**Published:** 2012-01-27

**Authors:** Agustín Sáez, Malena Sabatino, Marcelo A. Aizen

**Affiliations:** Laboratorio Ecotono-CRUB, Universidad Nacional del Comahue, San Carlos de Bariloche, Río Negro, Argentina; University of Lancaster, United Kingdom

## Abstract

Pollinators for animal pollinated crops can be provided by natural and semi-natural habitats, ranging from large vegetation remnants to small areas of non-crop land in an otherwise highly modified landscape. It is unknown, however, how different small- and large-scale habitat patches interact as pollinator sources. In the intensively managed Argentine Pampas, we studied the additive and interactive effects of large expanses (up to 2200 ha) of natural habitat, represented by untilled isolated “sierras”, and narrow (3–7 m wide) strips of semi-natural habitat, represented by field margins, as pollinator sources for sunflower (*Helianthus annus*). We estimated visitation rates by feral honey-bees, *Apis mellifera*, and native flower visitors (as a group) at 1, 5, 25, 50 and 100 m from a field margin in 17 sunflower fields 0–10 km distant from the nearest sierra. Honey-bees dominated the pollinator assemblage accounting for >90% of all visits to sunflower inflorescences. Honey-bee visitation was strongly affected by proximity to the sierras decreasing by about 70% in the most isolated fields. There was also a decline in honey-bee visitation with distance from the field margin, which was apparent with increasing field isolation, but undetected in fields nearby large expanses of natural habitat. The probability of observing a native visitor decreased with isolation from the sierras, but in other respects visitation by flower visitors other than honey-bees was mostly unaffected by the habitat factors assessed in this study. Overall, we found strong hierarchical and interactive effects between the study large and small-scale pollinator sources. These results emphasize the importance of preserving natural habitats and managing actively field verges in the absence of large remnants of natural habitat for improving pollinator services.

## Introduction

Animal-mediated pollination is one of the most critical processes involved in the reproduction of wild and cultivated flowering plants often limiting seed production [1], [2]. Because >70% of all agricultural crops depend to some extent on pollinators to maximize their yield [1], [3], the pollination service provided by flower visitors nesting or gathering food in neighboring natural or semi-natural habitats has an important role in global food production [4]. Several studies show that fruit and/or seed output of most pollinator-dependent crops is improved mainly by managed or feral honey-bees and secondly by wild bees that rely on resources provided by these ancillary habitats [5]–[12]. However, the destruction and fragmentation of natural or semi-natural habitat remnants through agricultural intensification are among the main causes of the decrease in local and global biodiversity in general [13], and pollinator abundance and diversity in particular [14], [15]. Habitat degradation can also be associated with the inadequate use of pesticides and herbicides, and introduction of alien species, which can also be important causes of pollinator decline [16]. Because many pollinators are far-ranging foragers but prefer to harvest resources locally, their demise can result from compound and interacting effects of habitat destruction and fragmentation occurring at different scales. As a consequence, an understanding of how different small- and large-scale habitat types contribute and interact to sustain vigorous bee populations is of paramount importance to their preservation and, through this mechanism, improve agricultural yield.

Bees require a nest to raise their brood, pollen to feed their larvae, and nectar to support their adult life [17]. Remnants of natural and semi-natural habitats in agricultural ecosystems usually provide abundant and diverse nesting sites and floral resources. In contrast, crop fields usually represent poor nesting habitats, while providing abundant but little-diversified and time-restricted floral resources [18]. Therefore, remnants of natural or semi-natural habitats usually sustain higher bee abundance and diversity than nearby cultivated fields [5]. Given these differences in habitat quality and the fact that most bees are central-place foragers (i.e., they fly from their nest sites to localized foraging sites), habitat fragments retaining native vegetation and field margins rich in agricultural weeds can become important pollinator sources for adjacent crops. However, the relative importance of these different habitat types as sources of pollinators and pollination service in agroecosystems will be ultimately determined by the size of the bee populations they can support and their distance to cultivated fields. Because typical bee foraging distances range from several hundreds meters to a few kilometers [19], [20], both local and regional pollinator sources can be relevant in determining the magnitude of this service. Here we investigated the relative importance and interactive effects of large remnants of native vegetation and weedy field margins in supplying pollinators, mainly feral honey-bees, for neighboring sunflower fields in the Argentine Pampas.

The Austral biogeographic district of the Pampas [21] constitutes one of the most intensively- and extensively-used agricultural landscapes in South America. Sunflower, *Helianthus annus*, is an important pollinator-dependent crops cultivated in the region. In agricultural settings around the world, this crop is almost exclusively visited and pollinated by both domesticated and feral honey-bees, which exploit sunflower for both nectar and pollen while improving its seed production and oil content [5], [6], [22]. The flat topography of southwestern Buenos Aires province, Argentina, is interrupted by a series of ancient (lower Paleozoic) eroded hills, “sierras”, jutting out of the loessic (Quaternary) Pampean plains. These sierras range in area from tens to thousands of hectares and reach a maximum height of a few hundred meters. Most of them remain untilled because of steep slopes, shallow soils and exposed bedrock, thus retaining much of the original shrubby native vegetation. Also, some cultivated fields are bounded by one or rarely more uncultivated margins, a few meters wide, which despite being frequently burned, mowed and grazed support diverse herbaceous communities of common agricultural alien weeds and some ruderal native flowering plants. Thus, whereas sierras represent high-quality, and field margins poor-quality nesting habitat, both sierras and field margins provide diverse and abundant floral resources for several species of bees, including feral honey-bees [23].

Our general hypothesis was that both sierras and field margins act as a source of pollinators, mostly feral honey-bees, for nearby sunflower fields. However, we expected an interaction between these two pollinator sources because of differences in the extent and quality of these different habitats [9], [24]. We view field margins mostly as “stepping-stone” habitats for honey-bees, defined here as small areas that become secondary pollinator sources because of recruiting foraging bees that reside elsewhere. Thus, we hypothesized that the sierra effect should neglect any field-margin effect for sunflower fields surrounded by nearby large expanses of natural, high-quality habitat, whereas a field-margin effect should be increasingly apparent for isolated sunflower fields. We focused here on pollinator visitation frequency, excluding a formal analysis on pollinator diversity, because of the dominance of honey bees as the almost exclusive sunflower visitor in our study area. Specifically, we tested the following predictions: (1) visitation frequency to sunflower increases with the amount of the sierra habitat neighboring a sunflower field, (2) visitation frequency to sunflower declines with distance from the field margin, and (3) this field-margin effect becomes increasingly apparent in sunflower fields far away from the sierras. Although several studies have reported habitat isolation and edge effects on pollinator diversity and abundance [12], [25]–[27], to our knowledge this is the first investigating the interaction of two sources differing in hierarchy and expected magnitude of their effects. Unveiling scale-dependent habitat interaction is important in guiding the design and management of agricultural mosaics.

## Materials and Methods

### Study area

**Figure 1:**
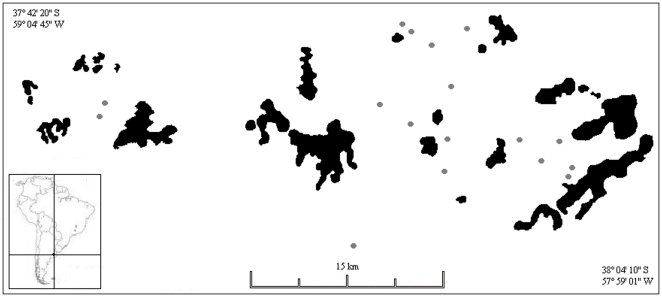
Location of the study region and sunflower fields. Study region and its geographic location in southern South America. Shown are the area occupied by the “sierras” (black color) and the location of the 17 study sunflower fields (grey color).

The study area is located nearby the city of Balcarce (37°50′S, 58°15′W). About 90% of this area is agricultural land dominated by soybean, sunflower, wheat, corn, potato, rape, and some scattered pastures. Apiculture with European varieties of *Apis mellifera*, the common honey-bee, is also an important economic activity in this agricultural matrix. Within this area, there are 24 isolated sierras, part of the Tandilia orographic system, ranging from 12 to 2200 ha (Figure 1) with maximum altitudes ranging between 400 and 500 m. The rocky terrain of these sierras supports diverse vegetation dominated by shrubs, herbs, and geophytes. Sunflower fields typically ranges from about 20 to 50 ha, and are cultivated with different sunflower hybrids. Cultivated lots are fenced and bounded on one or more sides by an uncultivated strip, 3–7 m wide, rich in alien agricultural weeds and native ruderal plants.

### Field sampling

Field work was conducted during the 2008–2009 sunflower blooming season (December–January). We sampled a total of 17 sunflower fields located 0 to 10 km away from the nearest sierras and bounded on at least one side by an uncultivated margin. These fields were all on Argiudoll soils [28], and lacked any domestic beekeeping activity within a radius of at least 1.5 km (A. Saez, personal observation). Although honey bees can forage several km from the hive, most common foraging distances are <2 km [20], [29]–[31]. Distances between surveyed fields ranged typically from 3 to 10 km, exceeding the expected flight distances of most foraging bees and other invertebrate flower-visiting taxa [19], [30], [32], [33]. In the few cases that the distance between nearest sunflower fields was less than 3 km, they did not overlap in flowering phenologies. Thus, each field can be considered an independent replicate in terms of their respective flower-visiting fauna. In each field, we observed insects visiting sunflower inflorescences (i.e., “heads”) at 1, 5, 25, 50 and 100 m from one of the margins, randomizing the order that these five distances were sampled and of sampling stations within distances. The maximum sampling distance, i.e. 100 m, was chosen based on the typical range of distances from the center of the fields to the nearest margin, being between one and a few hundred meters at the maximum. In each sampling station, we observed 7–15 focal heads during 10 min, counting and identifying all flower visitors and the number of flower heads visited by each insect. In our insect counts, we only considered flower visitors that made contact with anthers and/or stigmas. Identification of flower visitors in the field was carried out with the aid of a reference collection. Each field was surveyed on two different days over the flowering season, twice in the morning (between 9–12 hours) and twice in the afternoon (between 15–18 hours), sampling only during sunny or slightly cloudy days with low wind velocity. All necessary permits for field work were obtained through the National Institute of Agricultural Technology (INTA), Balcarce. Field locations are not protected in any way and this study does not involve any species listed as endangered or protected.

### Data analysis

For each focal sunflower field, we estimated its degree isolation from the sierra habitat considering both the area of the neighboring sierras and distance to those sierras. Among many different indices of habitat proximity [34], following Steffan-Dewenter et al. [35] we chose 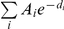, where *A_i_* was the area of the sierra *i* in hectares and *d_i_* the minimum distance in kilometers from the margin of the focal sunflower field to the edge of sierra *i*. For each focal field, we included all sierras *i* within a radius of 10 km, about the longest bee foraging distance [19], [35], [36]. We used ArcGIS v. 9.2 to determine the area of each sierra as well as the linear distance between each of them and each focal sunflower field. In our study system, the habitat proximity index varied from 0.6 (for a field located at 10 km from the nearest sierra) to 1948 (for a field at the foothill of a large sierra and surrounded by other sierras nearby) and had units of ha.

To evaluate the effects of isolation from the sierras, distance to the field margin, and the interaction between both factors on visitation frequency to sunflower heads, we used a generalized multilevel regression model fitted with the function lmer (library: lme4; [37]) of the statistical software R version 2.7.2 [38]. Multilevel regression analysis is based on a partial-pooling estimation of model parameters, following a hierarchical factor structure [39]. We used this approach to estimate the influence of isolation from the sierras, modeled as whole-plot effect, and the distance to the field margin and interaction between both habitat factors, modeled as within-plot effects. Because >90% of all sunflower visits were accounted by *Apis mellifera* (see Results), we analyzed separately the effect of habitat isolation and distance from the field margin on visitation by (1) honey-bees and (2) all other visitors, mostly represented by native insects, as a group (hereafter referred collectively as “native insects”). Numbers of flower heads visited were counts, so we assumed a *Poisson* error distribution and a *log* link function. We included number of sunflower heads observed in each census as an *offset*, i.e. a fixed predictor known in advance to influence insect visitation [39]. Visitation frequency, the output variable, was expressed as number of visits. flower head^−1^⋅10 min^−1^. Visits by native insects was also analyzed as a binary variable, 0 (absence) and 1 (presence) by means of a multilevel logistic regression model, because native insects were not observed in >50% of the censuses and they rarely visited >1 flower head when observed. Both dependent variables, isolation from the sierras and distance to the field margin, were log-transformed because they varied according to an exponential scale.

Following Gelman & Hill [39], we fitted models of increasing complexity. We first fitted a model where we analyzed only the whole-plot factor, i.e. isolation from the sierras. Secondly, we included the within-plot factor, i.e. distance to the field margin. Thirdly, we fitted the full model that included the large- and small-scale factors and the interaction between them. We used the Akaike's Information Criterion (AIC) to choose the best model [40].

## Results

We recorded a total of 2615 visits made by 1803 floral visitors. All flower-visitors were insects with the exception of one visit by the hummingbird, *Chlorostilbon aureoventris*. The common honey-bee, *Apis mellifera*, accounted for nearly 94% of all visits and was observed in 97.9% of all censuses. Native insects accounted for the remaining 6% of all visits and were observed in 35.8% of all censuses. Mean ± SE visitation frequency by honey-bees and native insect were 0.94±0.0026 and 0.06±0.003 visits⋅head^−1^⋅10 min^−1^, respectively. About half of all visits by native flower visitors were accounted by insects belonging to the orders Hymenoptera (1.7%), and Coleoptera (1.4%). Among native bees, we recorded 25 visits by *Melissoptila tandilensis*, four by *Xylocopa augustii*, and one by *Bombus bellicosus*. Visits by insects in the orders Diptera, Hemiptera and Lepidoptera each represented <1% of the total.

**Figure 2:**
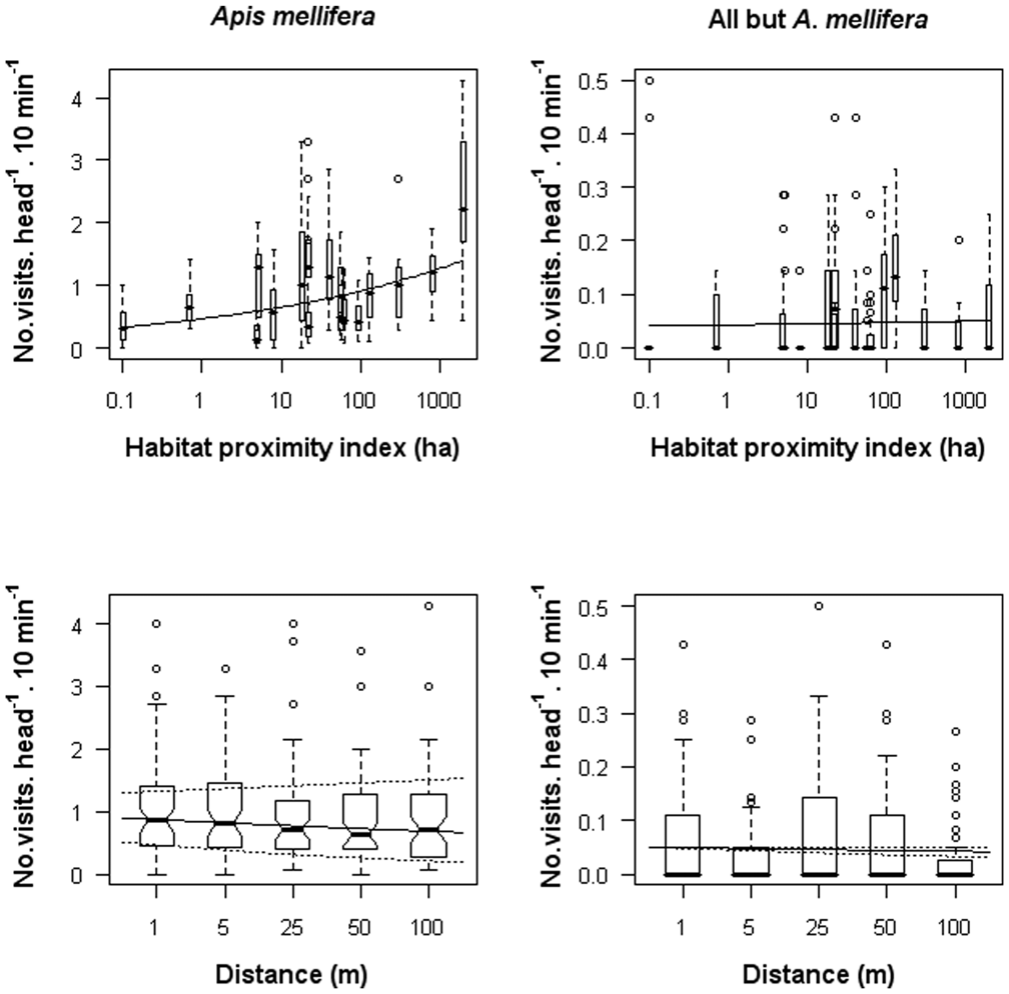
Effects of proximity to the sierras and distance to the field margins on visitation frequency. Box plots of frequencies of insect visits to sunflower heads in relation to proximity to the sierras (upper panels) and distance to the field margin (lower panels) for *Apis mellifera* (left panels) and “native insects” (right panels). Solid lines depict the predicted partial regression curves. In the lower panels, the lower and upper dotted curves depict the expected distance-to-the-margin effect for the most and least isolated sunflower field, respectively, based on the most complete generalized linear model that includes the interaction between the two habitat factors.

Increasing isolation from the sierras strongly decreased the frequency of visits by *Apis mellifera*. In the field closest to the largest sierras, visits by honey-bees were, on average, about four times as frequent as in the most isolated fields, decreasing from about 1.39 to 0.33 visits⋅head^−1^⋅10 min^−1^ (Figure 2). The whole-plot model had an AIC = 821.9 and revealed a significant proximity-to-sierra effect (estimate ± SE = 0.14±0.051, *z* = 2.71, *P*<0.01). Inclusion of the within-plot factor increased model fit (AIC = 812; i.e. ΔAIC<−2), showing that honey-bee visitation varied with distance from the field margin (−0.041±0.012, *z* = −3.46, *P*<0. 001). According to this model, visitation frequency was predicted to decrease by about 25%, from 0.93 to 0.70 visits⋅head^−1^⋅10 min^−1^, at 100 m from the field margin (Figure 2). However, the third model, including the interaction between the whole- and within-plot factors, still provided a better fit (AIC = 796.7), demonstrating that the direction and magnitude of the regression slope associated with the field-margin effect depended on the degree of isolation from the sierras (0.023±0.006, *z* = 4.13, *P*<0. 0001). Whereas a negative distance effect from the field edge on honey-bee visitation was not apparent in the least isolated fields from the sierras, the full model showed that the already low honey-bee visitation recorded in the most isolated fields further declined with distance from the field margin (Figure 2). In the field with the lowest habitat proximity index, for example, visitation frequency was predicted to decrease by 60% (from 0.57 to 0.22 visits⋅^−1^⋅10 min^−1^), comparing the field edge to 100 m. Thus, our hierarchical analysis revealed that proximity to the sierras and field margin, as well as the interaction between them, all influenced visitation by honey-bees.

**Figure 3:**
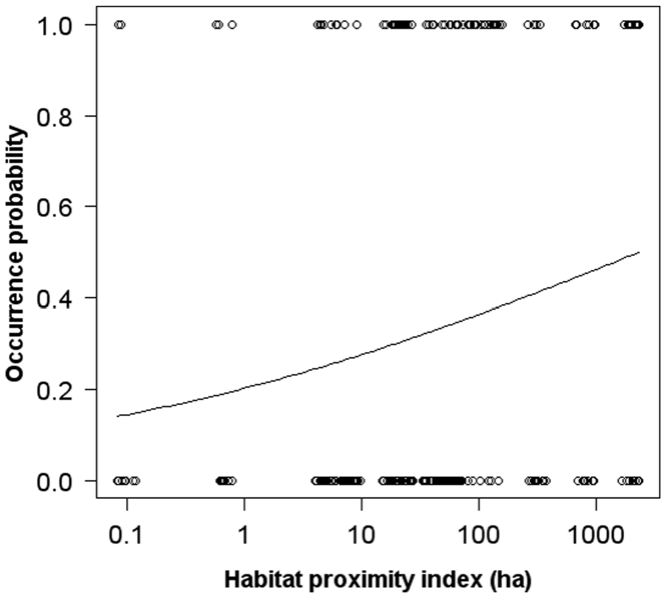
Changes in the probability of observing a native insect with proximity to the sierras. Estimated probability of observing a native insect visiting a sunflower head in relation to proximity to the “sierras”. The curve represents the fitted logistic regression (see Materials and Methods).

Visitation by all native insects lumped together was not explained by any of the two study habitat factors. The whole-plot model, including proximity to the sierras as the only explanatory habitat factor had an AIC = 343.1, and did not fit the data better than a model including only the intercept (AIC = 341.4). Similarly, a model including distance from the field margin as a within-plot factor did not improve fit (AIC = 344.3), and neither did the more complete model that additionally considered the interaction between isolation to the sierras and distance from the field margin (AIC = 346.3). In none of the models, none of the individual habitat factors explained any significant variation in visitation frequency by native visitors (*P*>0.10; Figure 2). Furthermore, there was no evidence that visitation rates by native insects and honey-bees covaried negatively overall (*r* = 0.08, *n* = 295, *P* = 0.16), as it could be expected if honey-bees competitively excluded native flower visitors. On the contrary, we found a trend towards a more positive correlation between visitation frequencies by honey-bees and native insects with increasing field isolation; the slope of the linear regression between (log) habitat proximity and the correlation coefficient estimated for each field was close to significance (−0.13+0.063, *F*_1,15_ = 4.23, *P* = 0.058). Despite none of the study habitat factors explained any variation in visitation frequency by native insects, a hierarchical logistic-regression model including proximity to the sierras as the only explanatory variable showed that the probability of observing a native insect visiting a sunflower flower head decreased marginally with increasing isolation (0.18+0.097, *z* = 1.82, *P* = 0. 068). The regression equation predicted that the probability of observing a native flower visitor in our censuses decreased from 0.49 to 0.14 from the least to the most isolated sunflower field (Figure 3). On the other hand, we did not find any evidence in the more complex models that distance to the field margin, or its interaction with isolation from the sierras, influenced significantly this probability (*P*>0.50).

## Discussion

Animal pollination is important for increasing yield of many agricultural crops [1], [3], [5], [9], [11], [12]. In this context, remnants of natural and semi-natural habitats can become sources of pollinators for adjacent cultivated fields. Indeed, declines in pollinator diversity and abundance with distance to these remnants, including edge effects on feral honey-bees, have now been reported for several crops [14]. However, to our knowledge ours is the first study demonstrating that local edge effects on pollinator visitation can be modulated by larger-scale regional effects. Particularly, we found that a decrease in visitation by feral honey-bees to sunflower heads occurring at a scale of 100 m from the field margins was not detected on the proximity of large expanses of natural habitats provided by sierras, whereas this local edge effect was apparent in sunflower fields located several kilometers away from the nearest sierra.

The honey-bee (*Apis mellifera*) was the overwhelmingly dominant flower visitor and presumed pollinator of sunflower in our study area, accounting for >90% of all visits and >95% of all bee visits. Similar results were reported by Torreta et al. [41] who worked in eight different agricultural areas of Argentina, including the Pampas. In California, honey-bees also dominated the pollinator assemblages surveyed in 16 sunflower fields [6]. However, in that region honey-bees accounted for a lower proportion (ca. 70%) of all bee visits, despite the study fields being stocked with domestic honey-bee hives. We do not know whether these differences in relative visitation frequency by honey-bees vs. native bees are specific to the Pampas *vs.* California comparison or are representative of a more general biogeographical contrast. We do know, however, that invasion by honey-bees, starting in 1956, has been widespread in South America [42], even in temperate regions like the Pampas and Patagonia [23], [43] where limited gene flow between African- and European-derived populations has been recorded [44]. In southern South America, feral honey-bees dominate now pollinator assemblages associated with many pollinator-dependent crops [12], [45] as well as pollinator-generalist plants growing in the wild [46]. Incidentally, the number of managed honey-bee hives has been declining in different parts of Argentina during the last years, largely due to parasitic mites (e.g., *Varroa destructor*), improper pesticide and herbicide use, and loss of floral biodiversity through agricultural intensification [47]. Thus, problems with domesticated honey-bees highlight the importance of natural and seminatural habitats as sources of pollinators in South America, even in the form of feral honey-bees [12], [48].

Although feral honey-bees can thrive in highly disturbed and fragmented habitats [15], crop fields provide poor nesting resources because of a lack of woody cavities or rocky cracks where to build a protected hive, or high standing trunks where to build a free-hanging hive [49]. In addition, crop fields can provide abundant but low-diversity floral resources that are available only for a short period, which can further limit the abundance of foraging honey-bees [18]. Hence, decreases in visitation frequency by feral honey-bees with distance to remnants of natural or semi-natural habitat, where feral honey-bees nest and/or forage more frequently, can be expected despite their opportunistic behavior and long-foraging ranges [14], [33], [50]. For instance, Chacoff & Aizen [12] found average declines of 50% in visitation frequency by feral honey-bees from the forest edge to 1000 m inside grapefruit plantations in NW Argentina, and Blanche et al. [51] reported three times more honey bees seen visiting macadamia flowers in orchards near (<0.5 km) than far (<4 km) from rainforest vegetation in Australia. Thus, the declines in visitation frequency we found here of up to about 70% for fields several kilometers away from the sierras, and average declines of 25% at 100 m from field margins are congruent with these previous results.

Differences in habitat extent and quality between the sierras and field margins, however, can lie behind the predicted and confirmed interaction between these two pollinator sources. Assessment of the area, distribution, and intrinsic value of each habitat type in terms of nesting and floral resources availability, as well as information on bee foraging distances can predict pollination services across agricultural landscapes [52]. In our study system, the sierras represent a habitat complex rich in nesting sites for honey-bees due to the presence of many cracked outcrops and hollowed stumps, and with abundant and diversified year-around floral resources associated with a highly endemic flora [53], [54]. Indeed, *Apis mellifera* was recorded as the most common flower visitor in the sierras dominating the pollinator assemblage of several native species and pollination webs [23]. Not surprisingly given their size and high habitat quality the sierras can be viewed as major providers of pollinators at the regional scale, here in the form of feral honey-bees, for neighboring sunflower fields and other crops. On the other hand, the linear and highly disturbed semi-natural habitat represented by the field margins is rich in flowering agricultural weeds and some ruderal native plants, but these margins are more limited in extent and quality than the sierras in terms of providing only poor nesting conditions for honey bees. However, without the presence of this semi-natural, highly-disturbed habitat, the incidence and abundance of wild bees in areas far away from the sierras would be greatly impaired. Thus, the large feral honey-bee populations spilling over the sierra boundaries should be determining high visitation frequency in sunflower fields neighboring these large expanses of high-quality habitat. According to our hypothesis, the strong effect associated with the large feral honey-bee populations from the sierras overshadows any effect coming from the smaller feral and transient honey-bee populations using floral resources in the semi-natural habitat bordering the fields. The more local field-edge effect should be only apparent for poorly-visited fields distant from the sierras. Indeed, field margin can be seen more as a stepping-stone habitat for feral honey-bees inhabiting the sierras or other unrecorded habitat. Our results clearly support this conceptual model.

Unlike honey-bees, native insects, including native bees, visited sunflower heads infrequently and their visitation rates apparently were not influenced by proximity to either the sierras or the field margins. It is difficult to explain this lack of effect because the sierras, and to a lesser extent the strips of semi-natural vegetation extending along field margins harbor rich and diverse pollinator communities [23]. Also, a meta-analysis showed general proof of a decline in native pollinator abundance and diversity with distance to remnants of natural and semi-natural habitats, and these trends were revealed as the most common and strongest pollination-related edge effects [14]. However, it is also true that several individual studies reported no evidence for edge effects on native flower visitors [14]. One possibility here is that strong resource or interference competition could exclude native insects from fields or patches heavily visited by honey-bees. Yet, we found no evidence for an overall negative association between visitation frequencies by honey bees *vs.* native insect as it might be expected under a competition scenario [25]. We cannot discard this hypothesis completely, however, because competition might be only occurring in the fields closest to the sierras where visitation rates by honey bees are the highest, while becoming diluted in the most isolated fields where even positive associations between honey-bees and native floral visitors were detected, perhaps mediated by a heterogeneous nectar and/or pollen resource distribution. A second possible explanation is based on the large variability associated with the highly sporadic and erratic visitation shown by native insects. Eventually, a pattern considering this response variable would require of a more intensive sampling to surface. Indeed, we detected evidence of a potential sierra effect, when large part of visitation variability was collapsed into a binary variable (Figure 3), thus providing an indication that the probability of observing a native floral visitor in a sunflower patch decays with isolation from the sierras. In any event, we can conclude that the lack of significant results does not obscure any significant biological pattern as native insects, and particularly native bees, represented a very minor component of the study pollinator assemblage.

The evidence of interactive effects between habitats differing in their strength as pollinator sources is important for managing agricultural landscapes. First, our results emphasize the importance of preserving natural habitats as major sources of pollinators, even of alien honey-bees. Regrettably, these sierras, among the last few remnants supporting Pampean biodiversity, are not acknowledged as warranting conservation status and most sierras in the Tandilia system are subject to frequent human disturbance. The view, supported by evidence here, that the sierras can supply different ecosystem services, including pollinators and crop-pollination, provides cause for their preservation. Second, our results also provide justification for actively managing marginal habitats along field verges in the absence of large remnants of natural habitat for improving pollinator services. For instance, in our case forage sites for feral honey-bees and also nesting sites for other bees could be added to this habitat, and frequency and timing of burning could be implemented in a way as to maximize encroachment of flowering weeds [55]. Thus, understanding the hierarchy and interaction of different pollinator sources can lead to a better management and understanding of the agricultural landscape.
